# Using non-insecticidal traps indoors can complement insecticide-treated nets to target insecticide-resistant malaria vectors

**DOI:** 10.1186/s13071-025-06759-2

**Published:** 2025-05-09

**Authors:** Romaric Akoton, Pierre Marie Sovegnon, Oswald Y. Djihinto, Adandé A. Medjigbodo, Romuald Agonhossou, Ayola Akim Adegnika, Gabriella Gibson, Rousseau Djouaka, Frances M. Hawkes, Luc S. Djogbénou

**Affiliations:** 1 Tropical Infectious Diseases Research Center (TIDRC), University of Abomey-Calavi, Cotonou, Benin; 2 Fondation Pour la Recherche Scientifique (FORS), Cotonou, Bénin; 3 International Institute of Tropical Agriculture, Cotonou, Benin; 4 Centre de Recherches Médicales de Lambaréné (CERMEL), Lambaréné, Gabon; 5 Institute for Tropical Medicine (ITM), University of Tübingen, Tübingen, Germany; 6 Department of Agriculture, Health & Environment, Natural Resources Institute (NRI), University of Greenwich at Medway, Kent, UK; 7 Regional Institute of Public Health, University of Abomey-Calavi, Ouidah, Benin; 8 German Center for Infection Research (DZIF), partner site Tübingen, Tübingen, Germany

**Keywords:** Host decoy trap, Pyrethroid resistance, Insecticide-treated nets, Vector control, *Anopheles gambiae s.l.*

## Abstract

**Background:**

Insecticide-treated nets (ITNs) provide protection against malaria vectors through their insecticidal action and as a physical barrier. However, insecticide resistance in malaria vectors has diminished their efficacy, threatening future malaria control. To reinforce ITNs’ effectiveness, evaluating non-insecticide-based tools in an integrated control approach is worthwhile. In the present study, a mosquito collection technique, the Host Decoy Trap (HDT), was coupled with standard ITNs as a complementary intervention, and its effectiveness against insecticide-resistant *Anopheles gambiae* s.l. was assessed in experimental huts.

**Methods:**

An HDT combined with either permethrin or deltamethrin-treated nets was tested against field-collected *An. gambiae* mosquitoes from Za-Kpota (Benin Republic) in experimental hut trials following WHO Phase II guidelines. Effectiveness was assessed in terms of mosquito mortality, blood feeding and exophily rates. Prior to hut trials, an insecticide susceptibility test was performed on field-collected *An. gambiae* s.l. mosquitoes to screen for pyrethroid resistance.

**Results:**

A significantly higher mortality rate was observed against both susceptible and field-collected *An. gambiae* s.l. mosquitoes when ITNs were used with HDT (ranging from 80.18 to 99.78%) compared to alone (2.44–100%). The combined use of treated nets with HDT resulted in a lower rate (ranging from 0 to 10.83%) of blood feeding compared to the treated nets alone (ranging from 0 to 16.93%). When treated nets were hung next to the HDT, they significantly limited the number of insecticide-resistant mosquitoes that exited experimental huts compared to the nets alone.

**Conclusions:**

The use of HDT alongside ITNs has been demonstrated to significantly reduce the likelihood of vector-host contact by insecticide-resistant *An. gambiae*. A combination of HDT and treated nets reduced the number of live *An. gambiae* mosquitoes as well as the blood-feeding rate. Furthermore, it reduced the number of mosquitoes likely to leave the huts and enter the natural environment. Altogether, our findings highlight the potential of integrated approaches combining non-insecticidal trapping devices with ITNs when designing future integrated vector control strategies.

**Supplementary Information:**

The online version contains supplementary material available at 10.1186/s13071-025-06759-2.

## Background

The continued use of insecticide-treated nets (ITNs) represents a core strategy against malaria transmission in endemic countries [1, 2]. Unfortunately, the growing phenomenon of vector resistance to existing insecticide products has led to a decline in the effectiveness of ITNs in reducing malaria prevention [3–5]. Many studies in areas of high vector resistance have revealed a decline in the efficacy of ITNs [3–7]. A recent meta-analysis of data from bioassay and experimental hut studies showed that community protection provided by these existing ITNs falls rapidly as insecticide resistance emerges [8]. According to World Health Organization (WHO) recommendations, exploring novel vector control strategies, comprising a mixture of non-related insecticides, is imperative to ensure the success of insecticide resistance management strategies [9, 10]. The efficacy of new ITNs combining pyrethroids and piperonyl butoxide (PBO) has been demonstrated in laboratory and field trials against pyrethroid-resistant malaria vectors [5, 11–15]. Despite the recent recommendation for their deployment in areas where the main malaria vectors exhibit metabolic resistance mediated by cytochrome P450s monooxygenases [16, 17], there is still uncertainty regarding long-term effectiveness of pyrethroid-PBO ITNs [6]. Recent studies have indicated that the pyrethroid-PBO-based nets are also becoming less effective [6, 18]. Two additional new-generation nets, Royal Guard and Interceptor G2, have also shown significant efficacy against pyrethroid-resistant *Anopheles gambiae* s.l., making them potential cornerstones in future malaria prevention [19, 20].

Although these new generation nets represent an important contribution to vector control, a resistance phenomenon against insecticides is an evolutionary adaptation that can not only shorten the lifespan of currently available insecticide-based tools but ultimately undermine the longevity of newly developed insecticides, too [21]. Considering the recent history of the decline in the effectiveness of pyrethroid-PBO-based nets [6, 18], it is reasonable to question the long-term performance of these new-generation nets also. A study conducted in Burkina Faso highlighted that resistance still led to decreased mosquito mortality 24 h post-exposure to Interceptor G2, suggesting a possible reduction in the overall effectiveness of this net in areas with significant pyrethroid resistance [22]. Moreover, the limited ability of currently deployed tools to control insecticide-resistant vectors highlights the integration of non-insecticide-based tools in vector control as a potential long-term approach to sustain the efficacy of current and incoming ITNs against malaria vectors [7].

Recently, there has been considerable innovation in mosquito sampling tools, several of which show considerable potential as non-insecticidal trapping devices with which to diversify the methods used to control malaria vectors and, ultimately, prevent malaria cases [23, 24]. The Host Decoy Trap is one such device, which employs a combination of human-associated stimuli, including olfactory, visual and thermal cues, to lure and kill host-seeking female mosquitoes [25]. Compared to the gold standard human landing catch (HLC) tool, HDT was found to be able to trap up to ten times the number of *Anopheles* mosquitoes [25] and has been suggested as a potential candidate for operational use [26]. However, it has yet to be tested as a possible vector control tool. Due to its host-mimicking properties, deploying HDT in combination with bednets warrants investigation as a possible strategy for reducing the number of mosquitoes attempting to bite the host under the net by luring away and killing mosquitoes without using insecticides. Herein, we report entomological evidence that an integrated system combining HDT with pyrethroid-treated nets significantly increases the mortality of both susceptible and resistant *An. gambiae* s.l. mosquitoes.

## Methods

### Study site

Release/recapture experiments were carried out in experimental hut trials (EHTs) at the field station of the Tropical Infectious Diseases Research Center (TIDRC) of the University of Abomey-Calavi located in Ganhoua village in Za-Kpota district (07°10′58.4″ N, 002°17′15.3″ E), Southern Benin. In Za-Kpota, malaria transmission is perennial. Rice and vegetable production represent the primary agricultural activities and provide optimal breeding habitats for *An. gambiae* mosquitoes; there is significant pesticide use to protect the agricultural products [27].

### Mosquito collection and insecticide susceptibility assays

*Anopheles* mosquito larvae and pupae were collected from breeding sites near the experimental hut station at Za-Kpota between June and July 2023 using the dipping method [28]. They were transferred in labeled plastic bottles to an insectary and reared until the adult stage for insecticide-susceptibility testing. Mosquitoes were maintained under standard insectary conditions with a relative humidity of 70 ± 8%, an ambient temperature of 27 ± 2 °C and a 12:12 light and dark period. The insecticide susceptibility profile of 3–5-day-old F0 adult females reared from field-collected *An. gambiae* s.l. were assessed according to WHO tube test protocol [29]. Briefly, mosquitoes were exposed to filter papers impregnated with 0.05% alpha-cypermethrin, 0.75% permethrin and 0.05% deltamethrin (pyrethroids). Approximately 25 female mosquitoes were introduced into each of six tubes lined with either insecticide-impregnated paper (test tubes) or non-impregnated papers (control tubes). For each insecticide susceptibility assay, 150 female mosquitoes were used, divided into four test tubes (100 mosquitoes) and two control tubes (50 mosquitoes). The mortality was recorded 24 h post-exposure.

### Experimental hut trials

#### Mosquito strains

An insecticide-susceptible laboratory reference strain of *An. gambiae* s.s. (Kisumu), originating from Kenya, was maintained in the insectary [30]. The F0 adults of field-collected *An. gambiae* s.l. were obtained from larval collections conducted in the Za-Kpota district as described above.

#### Description of treatments used in experimental hut trials

The standard HDT was prepared using the methodology previously described by Abong’o et al. [31]. The trap consists of a bucket fitted with an external black cloth, which provides a visual cue and is wrapped with a transparent adhesive plastic sheet (Barrettine Environmental Health, UK) to catch mosquitoes as they land. A watertight bag containing approximately 15 l water heated to approximately 80 °C as measured using an infrared thermometer (IF-710-EUR) is placed inside the bucket. The water temperature inside the bag is sufficient to maintain a surface temperature across the cloth of 35 ± 5 °C for at least 12 h. This component of the standard HDT, designated the “HDT bucket unit”, provides high-contrast visual stimuli and human-equivalent thermal stimuli to induce close-range attraction and landing behaviour in host-seeking mosquitoes. In previous studies where the HDT was used outdoors, an olfactory cue was provided by a person resting in a small tent, whose odour was mechanically pumped towards the bucket and released within a few centimetres of its base [25, 31]; however, the present experiment took place inside experimental huts, and the HDT bucket unit, hereafter “HDT” in this study, was used without the tent odour component. Instead, a person sleeping on a bed under a bednet provided the source of human odour to stimulate host-seeking behaviour. The HDT was positioned 50 cm from the head of the bed. The bednets were standard pyrethroid-based nets in EHTs, arranged as shown in Additional file 1.

Six net treatments were tested in the EHTs. All the bednets were 180 cm long × 170 cm wide × 170 cm high. The specifications of each bednet are described in Table 1.

**Table 1:** Specifications of bednets tested in experimental hut trials

No.	Treatments	Abbreviation	Bednet manufacturer	Net fabric type and weave	Active ingredient doses
1	Untreated Net	UTN	Bayer AG, Leverkusen, Germany	Polyester fabric (100 mesh size)	No insecticide product
2	Untreated Net + HDT	UTN + HDT			
3	Olyset net	OS	Sumitomo Chemical, Tokyo, Japan	Polyethylene fibers (150 mesh size)	Permethrin at 20 g/kg
4	Olyset net + HDT	OS + HDT			
5	PermaNet 2.0	P2	Vestergaard Frandsen, Lausanne, Switzerland	Polyester fabric (100 mesh size)	Deltamethrin at 1.4 g/kg
6	PermaNet 2.0 + HDT	P2 + HDT			

#### Release/recapture of field-collected *An. gambiae* mosquitoes

Before the release/recapture experiments, six volunteers provided informed consent to participate in the study. Awareness sessions were conducted with the head of Ganhoua village and volunteers (to sleep under the bednets) to explain the study’s objectives. To reduce daily variability in olfactory products from volunteers, no consumption of beer was permitted during the study [32]. The volunteers also refrained from using soap, fragrance and repellent products applied on their body and did not use tobacco for 12 h before and throughout the testing period.

The three different types of bednet (UTN, OS, P2) were hung in the huts either alongside an HDT or without an HDT. The bednets were deliberately punctured with six holes of 16 cm^2^ (4 × 4 cm) following WHO guidelines [33] to simulate a damaged net as might be expected following long-term use or accidental damage. Prior to their release into the hut, all mosquito strains used were starved for 24 h with access to cotton soaked with water only. Release/recapture experiments were carried out according to the WHO protocol [33]. Briefly, each night (20:00 h), 100 adult female mosquitoes, 5 days old, were released in each hut and monitored until morning (05:30 h). In the morning, released mosquitoes were recaptured and their point of recapture noted (from within the hut, on the HDT, inside the nets or from the veranda). They were scored as dead or alive and as blood-fed or unfed. Five replicates were performed for each of the six treatments, and this design was carried out for *An. gambiae* s.l. from wild-caught larvae and was repeated in the same way using the susceptible laboratory Kisumu strain, making a total of 500 released mosquitoes per strain per treatment to ensure an adequate sample size. The six volunteer sleepers were allocated to the six treatments following a randomized Latin square experimental design to avoid any attractiveness and sampling bias. The recovery rate was between 89 and 95% across all treatments (Table 2); as we could not be certain of the outcomes for the small number of non-recovered mosquitoes, the following entomological parameters [33] were calculated as proportions relative to the total number of mosquitoes recaptured.

- Twenty-four-hour mortality rate: the proportion of mosquitoes killed in 24 h relative to the total number recaptured;
- HDT mortality rate: the number of mosquitoes recorded dead from the HDT as a proportion of all recaptured dead mosquitoes (24-h mortality rate);
- Blood-feeding rate: the number of mosquitoes collected and found to be blood-fed as a proportion of all mosquitoes recaptured;
- Exophily rate: the number of mosquitoes collected from the verandas as a proportion of all mosquitoes recaptured.

**Table 2:** Mosquito recapture rates across all treatments

No.	Treatments	Kisumu			Wild *Anopheles gambiae s.l.*		
		Total released	Total recaptured	Percentage ± standard error (SE)	Total released	Total recaptured	Percentage ± standard error (SE)
1	Untreated net	500	449	89.8 ± 3.25	500	450	90.0 ± 1.76
2	Untreated net + HDT	500	471	94.2 ± 1.39	500	459	91.8 ± 1.36
3	PermaNet 2.0	500	456	91.2 ± 2.89	500	469	93.8 ± 1.36
4	PermaNet 2.0 + HDT	500	454	90.8 ± 2.96	500	466	93.2 ± 0.37
5	Olyset net	500	444	88.8 ± 3.44	500	451	90.2 ± 0.37
6	Olyset net + HDT	500	471	94.2 ± 1.02	500	475	95.0 ± 1.10
Total		3000	2745		3000	2770	

### Molecular species identification of field-collected mosquitoes

To determine species composition of field-collected *An. gambiae* s.l. mosquitoes used in EHTs, a subset of approximately 40 alive and 40 dead females per treatment were identified molecularly using the protocol described by Santolamazza et al. [34].

### Data analysis

Data were recorded and entered into Microsoft Excel and were analyzed using R Statistical software version 4.3.0 [35].

Susceptibility test results (24-h mortality) were interpreted following the WHO criteria [29]. When mortality was ≥ 98%, the mosquito population is considered susceptible to the insecticide; a mortality rate between 90 and 97% implies suspected resistance, and < 90% indicates confirmed resistance to the insecticide. Since no mortality was recorded in controls, Abbott’s formula was not applied to correct the mortality rates.

All parameters from EHTs were analysed using a generalized linear mixed model (GLMM) with a logit link and binomial distribution. Mortality data recorded in all strains were analyzed using GLMM with a beta-binomial distribution. All models were fitted with sleepers (volunteers) and nights as random effects and treatment as fixed effects using the lme4 package [36]. The final models were selected according to the Akaike information criterion (AIC). Model fitting was evaluated by performing a quantile test, uniformity test and dispersion test using the DHARMa R package [37]. Two levels of comparison were determined for the entomological parameters: (i) Net + HDT vs Net alone; (ii) UTN + HDT vs ITNs + HDT. Ninety-five per cent confidence intervals (95% CIs) of adjusted odds ratio (ORs) were also determined using a suitable regression model by computing pairwise marginal means separately for each suborder, adjusting the *p* value using the Tukey method for multiple comparisons (package emmeans) [38]. All analyses were set at significance threshold of *p* < 0.05.

## Results

### Species composition of field-collected *An. gambiae* s.l.

Of the 2770 recaptured females of field-collected *An. gambiae* s.l. specimens from experimental hut trials, 415 (14.9% of recaptured mosquitoes) were subjected to molecular species identification. *Anopheles coluzzii* (90.78%) was the predominant sibling species in this mosquito population followed by *An. gambiae* s.s. (4.69%) and hybrids (4.53%).

### Insecticide resistance profile of field-collected *An. gambiae* s.l.

The field-collected *An. gambiae* s.l. were resistant to all insecticides tested according to WHO criteria. Specifically, they were resistant to permethrin, alpha-cypermethrin and deltamethrin with mortality rates of 26.2 ± 6.59%, 89.8 ± 0.86% and 54.25 ± 8.98%, respectively. No mortality was recorded in the control.

### Twenty-four-hour mortality rates in experimental hut trials

**Fig. 1:**
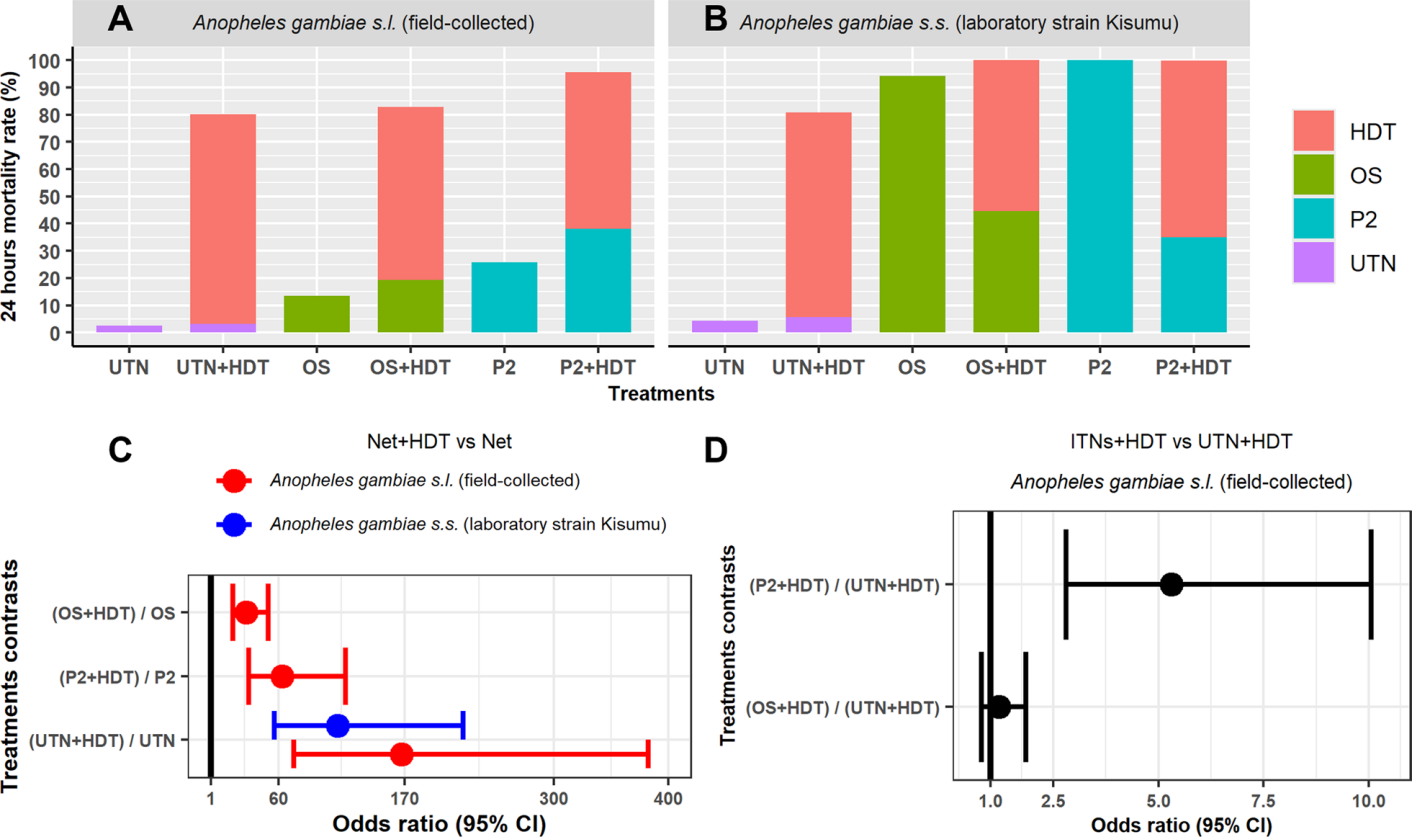
Proportion of killed mosquitoes according to (**A**) treatment and field *Anopheles gambiae* s.l., (**B**) treatment and Kisumu. Comparative odds ratio (OR) of mortality rate (**C**) between Net + HDT and Net alone and (**D**) between UTN + HDT and ITNs + HDT. Error bars in (**C**) and (**D**) represent 95% confidence intervals. *P2* PermaNet 2.0, *OS* Olyset net, *UTN* untreated net, *HDT* (Host decoy trap_bucket unit), *ITNs* insecticide-treated nets

Overall, the treatments combining ITNs and HDT displayed significantly higher mortality compared to those without HDT for both the laboratory strain of *An. gambiae* s.s. (Kisumu) (*df* = 1, *N* = 915, *χ*^2^ = 28.351, *p* < 0.0001) and field-collected *An. gambiae* s.l. mosquitoes (*df* = 1, *N* = 1825, *χ*^2^ = 295.040, *p* < 0.0001) (Fig. 1A–C).

Mortality rates recorded in the field-collected *An. gambiae* s.l. varied from 80.18 to 95.5% in the presence of HDT + ITNs systems. In these combinations, HDT alone lured and killed > 57% of released mosquitoes with 76.91% mortality rate in UTN + HDT, 63.58% in OS + HDT and 57.3% in P2 + HDT (*df* = 1, *N* = 925, *χ*^2^ = 27.033, *p* < 0.0001) (Fig. 1A). Compared to UTN + HDT, mortality in field-collected *An. gambiae* s.l. mosquitoes was significantly higher (5.3 times) with P2 + HDT (OR = 5.3; CI 2.81–10.05; *p* < 0.0010) (Fig. 1D). However, no significant difference was observed with OS + HDT (OR = 1.21; CI 0.79–1.85; *p* = 0.6718) compared to UTN + HDT (Fig. 1D).

With the susceptible Kisumu mosquitoes, HDT + ITNs induced mortality ranging from 80.89 to 99.78%. The HDT itself was responsible for > 55% mosquito mortality in the presence of all net types: UTN (HDT mortality rate 75.16%), OS (HDT mortality rate 55.41%) and P2 (HDT mortality rate 64.76%) (*df* = 1, *N* = 942, *χ*^2^ = 26.372, *p* < 0.0001) (Fig. 1B). HDT displayed no differential attractiveness to either susceptible or field-collected *An. gambiae* s.l. mosquitoes (OR = 1.10; CI 0.79–1.53; *p* = 0.5704) in the presence of UTN.

### Blood-feeding rate

**Fig. 2:**
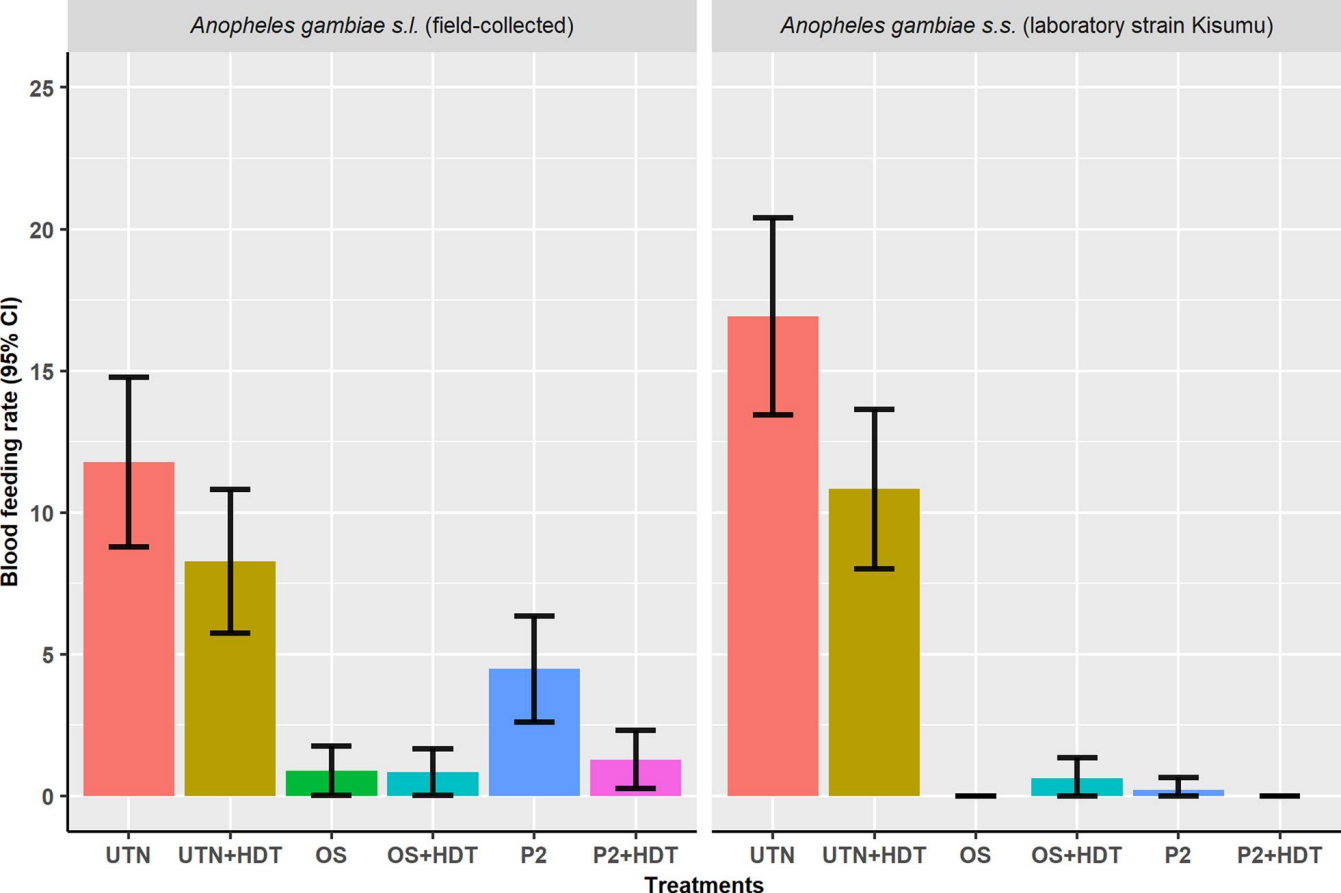
Proportion of blood-fed mosquitoes according to treatment. Error bars represent 95% confidence intervals. *P2* PermaNet 2.0, *OS* Olyset net, *UTN* untreated net, *HDT* (Host decoy trap_bucket unit), *ITNs* insecticide-treated nets

In the experimental hut trial, it was observed that the very low proportion of mosquitoes able to blood-feed was especially pronounced when HDT was combined with ITNs (Fig. 2), although blood-feeding rates were too low to enable statistical analysis. In field-collected *An. gambiae* s.l., a reduction in the proportion of blood-fed mosquitoes was observed when UTN was combined with HDT (8.28%; CI 6.20–10.89) compared to UTN alone (11.78%; CI 9.12–14.59). Only 1.29% (CI 0.56–2.66) of field-collected *An. gambiae* s.l. were blood-fed when exposed to PermaNet 2.0 in the presence of HDT (vs. 4.48%; CI 2.74–6.20 with P2 alone). In the conditions where the Olyset net was combined with HDT (OS + HDT), the proportion of blood-fed mosquitoes recorded was very similar (0.84%; CI 0.21–1.90 with OS + HDT vs. 0.89%; CI 0.22–1.99 with OS alone). The addition of an HDT also decreased the blood-feeding rate in the susceptible Kisumu strain when a UTN was present, from 16.93% (CI 12.63–18.79) to 10.83% (CI 7.87–12.90); this parameter was not discernibly different when treated nets were tested (ranging from 0 to 0.5%; CI − 0.07–1.13).

### Exophily rate

In Kisumu strain, the treatments combining P2 + HDT (5%; CI 2.69, 6.56) and OS + HDT (5.30%; 3.27, 7.34) displayed significantly lower exiting rates compared to the treated nets alone (OR = 0.14, CI 0.07–0.26, *p* < 0.001 for P2; and OR = 0.32, CI 0.17–0.59, *p* < 0.001 for OS) (Table 3). The same trend was observed in field-collected resistant *An. gambiae* s.l. when the treated nets were hung next to HDTs (8%; CI 5.29, 10.16 for P2 + HDT and 5.47%; CI 3.42, 7.53 for OS + HDT) compared to the treated nets alone (OR = 0.15, CI 0.09–0.24, *p* < 0.001 for P2; and OR = 0.12, CI 0.07–0.21, *p* < 0.001 for OS) (Table 3). Exophily rates were also reduced with UTN alongside HDT compared to UTN alone, although this was only significant in the Kisumu strain (OR = 0.27, CI 0.17–0.45, *p* < 0.001).

**Table 3:** Mosquito exit rates and comparatives odds ratio (OR) according to treatments

Treatments	Exophily rate (95% CI)	(Net + HDT) vs net	
		Odds ratio (95% CI)	*P* value
Kisumu			
Untreated net	26.06 (21.98, 30.13)	–	–
Untreated net + HDT	8.92 (6.33, 11.50)	0.27 (0.17, 0.45)	< 0.001*
PermaNet 2.0	25.88 (21.84, 29.91)	–	–
PermaNet 2.0 + HDT	5 (2.69, 6.56)	0.14 (0.07, 0.26)	< 0.001*
Olyset net	15.09 (11.75, 18.43)	–	–
Olyset net + HDT	5.30 (3.27, 7.34)	0.32 (0.17, 0.59)	< 0.001*
*Anopheles gambiae* s.l			
Untreated net	16.44 (13.00, 19.88)		
Untreated net + HDT	13.29(10.17, 16.41)	0.78 (0.48, 1.25)	0.5178
PermaNet 2.0	35.82 (31.46, 40.18)		
PermaNet 2.0 + HDT	8 (5.29, 10.16)	0.15 (0.09, 0.24)	< 0.001*
Olyset net	32.60 (28.25, 36.94)		
Olyset net + HDT	5.47 (3.42, 7.53)	0.12 (0.07, 0.21)	< 0.001*

*CI* confidence intervals

^*^Significance of difference

## Discussion

This study presents evidence that using the Host Decoy Trap (HDT) indoors in combination with pyrethroid-only nets may enhance the efficacy of such vector control strategies in the context of growing insecticide resistance. Vector mortality was enhanced when HDTs were used alongside widely used standard bednets (Olyset net and PermaNet 2.0) in both pyrethroid-resistant and laboratory-susceptible populations of the major malaria vector *An. gambiae* s.l. However, these standard bednets have lost much of their efficacy against resistant populations, as shown in previous studies [3–5, 39]. A significant proportion of observed mortality in field-caught resistant mosquitoes was attributable to the HDT (ranging from 57.3 to 76.91%), bringing total mortality in resistant populations to > 80% across all net types. We establish the principle of an integrated approach that combines conventional indoor vector control with a non-insecticidal lure and kill device, providing an important complement for future paradigms of vector control and resistance management.

In this study, a large number of Kisumu strain mosquitoes were lured and killed by the HDT, even when untreated bednets were used; this suggests that many host-seeking mosquitoes are attracted to and land on the HDT, regardless of the presence of a real human host beneath a nearby bednet. Although the device in this study was tested indoors, this finding highlights the earlier capture and subsequent mortality of *Anopheles* and other mosquito genera by the HDT when employed as an outdoor surveillance tool [25, 26, 31, 40]. The aforementioned studies indicate that the combination of human host-associated stimuli used in the HDT’s design are key factors in its effectiveness. The relative importance of different host stimuli has since been quantified [41, 42] and results from the present study suggest the visual and thermal stimuli arising from the HDT itself, in the presence of and in direct competition with a live host, are sufficient to lure and possibly deflect host-seeking vectors, as also indicated by the modest reduction in blood-feeding rates.

Interestingly, the mortality rate of resistant mosquitoes that was attributable to ITNs was also slightly increased when an HDT was present in the experimental hut. One explanation for this may be that host-seeking behaviour in general was enhanced by the additional stimuli arising from the HDT, resulting in more exposure to insecticides from more frequent contact with the ITNs. Although net contact times are generally lower on ITNs [43], recent research shows that permethrin causes some degree of increased persistence in host-seeking, measured as increased passage through holes in bednets in both resistant and susceptible mosquito populations [44]. Direct observation of mosquitoes and transcriptomic analysis could enhance understanding of their behavioural interactions with bednets, hosts (protected or not) and additional trapping/control devices in an indoor environment.

In experimental hut trials (EHTs), the HDT used consisted of only the visual and thermal elements of the trap itself, with odour and carbon dioxide emanating from the sleeping volunteer inside the bednet. Across all six treatments where an HDT was present, approximately 65% of mosquitoes were caught by the trap, indicating that this ambient odour source was sufficient to trigger host-seeking behaviour within the confines of a hut, while the cues from the trap prompted landing. The fact that the mosquito net itself provides a physical barrier likely enhanced the lure-and-kill properties of HDT. Placing a mosquito trap within a room used for sleeping may offer a more practicable option for limiting vector contact. This approach could be easier to implement compared to other suggestions that also exploit vector behaviour, for instance raising buildings to avoid low-flying mosquitoes [45], which may be challenging to implement in existing housing stock. In addition to malaria vectors, HDTs may also be useful against others nuisance mosquitoes such as *Mansonia* and *Culex* species and vectors of neglected tropical diseases [40]. Recently, it was highlighted that HDT may have a role to play in surveillance of *Simulium* vectors of *Onchocerca volvulus* in elimination settings [46]; given the extremely limited options for controlling adult blackflies, its use as a possible control tool may be worth exploring.

Further to the observed high killing effect against resistant mosquitoes, HDT contributed to the inhibition of blood-feeding in this study. Although only small numbers of blood fed mosquitoes were recorded in the huts, all the treatments combining nets and HDT reduced blood feeding rates compared to nets alone, probably by reducing the overall number of mosquitoes in the hut rather than altering their propensity to blood-feed. Pyrethroids are known to reduce both blood-feeding events and total blood volume ingested [47]; this finding suggests that overall a reduction in the risk of malaria transmission could be achieved by intercepting mosquitoes domestically before they have a chance to blood-feed, crucially, for both insecticide-susceptible and -resistant populations. Interestingly, none of the recorded blood-fed mosquitoes were lured and killed by HDT in the presence of ITNs in this study. As a key parameter influencing malaria transmission [47], the potential to reduce blood feeding requires further investigations, especially regarding at which point blood-feeding is prevented in a given household.

In this study, the high proportion of mosquitoes killed by HDT led to a significant reduction in the number of mosquitoes that could potentially escape the insecticide-based tools, leave the insecticide environments and enter the natural population where they could maintain the transmission of *Plasmodium* spp. and generate new offspring. It was reported that such tools that induce more exiting could reduce the chances of leading to a mass killing of the vector [48]. Significantly lower exiting rates were observed in both susceptible and resistant mosquitoes compared to the treated nets alone. Rather than perceiving this low exophily rate as a reduction in the repellency properties of treated bednets [6, 7, 49], it could be related to the high killing effect of HDT, thus removing mosquitoes before they can exit. This could be beneficial for malaria control. The observed low mosquito exophily could reduce the chances of them leaving the protected host environment, which would prevent them from continuing their life cycle. Moreover, the reduction in the number of mosquitoes likely to escape the insecticide environment could result in a decrease in the outdoor biting rate in the community, which would in turn lead to a reduction in residual malaria transmission. This was also highlighted in other published reports stating that high exiting rates could be in response to a wide-application indoor-based intervention, and this might contribute to residual malaria transmission [50, 51]. Moreover, in this study, HDT displayed no differential attractiveness to either susceptible or field-collected *An. gambiae* s.l. mosquitoes in the presence of an untreated net, suggesting that HDT would not show selective effectiveness according to insecticide resistance status in malaria vectors in environments where vector control interventions are absent, like outside households. This makes it a potential vector control tool to support sustenance of gains in control of residual malaria transmission. It can also function as a sampling tool that is not biased by resistance status of the target population.

The aforementioned data indicate that much attention should be paid to approaches that target vector host-seeking behaviour in combination with ITNs rather than solely on insecticide-based tools within houses. This research also opens perspectives on studying the epidemiological impact of HDT in endemic areas. In terms of potential large-scale applications, this approach could be enhanced by design modifications to increase lure/kill efficiency of HDT and make them easy to use without compromising efficacy. Consequently, it would be necessary to investigate communities’ perception regarding the system’s acceptability, practical use and perceived side effects. Combining bednets with HDT appears to be a promising mosquito control approach that could be integrated into current vector control strategies to tackle insecticide resistance.

## Conclusions

Suitable complementary tools for managing resistant malaria vectors are needed to reach malaria elimination in endemic settings. This study showed evidence that using a recently developed trap, the HDT, could reduce the chance of mosquito bites by significantly reducing mosquito survival and therefore contact with humans within the home and upon exit. The combination of HDT and bednets in the same room showed the capacity to reduce *An. gambiae* s.l. blood meal success and exophily rates. Our study sheds light on a potential integrated approach to combat resistant malaria vectors, which will be of interest to national malaria control programmes and others malaria control stakeholders.

## Supplementary Information

Additional file 1: Experimental set-up schematic of HDT with person under a bednet inside an experimental hut.

## Data Availability

All data generated or analysed during this study are included in this published article.

## Abbreviations

IITA: International Institute for Tropical Agriculture
ITNs: Insecticide-treated nets
OR: Odds ratio
PBO: Piperonyl butoxide
WHO: World Health Organization
