# Supplementary figures and images for: Using non-insecticidal traps indoors can complement insecticide-treated nets to target insecticide-resistant malaria vectors
Source: Parasit Vectors. 2025 May 9;18:166. doi: 10.1186/s13071-025-06759-2 (PMC12063245; doi:10.1186/s13071-025-06759-2)

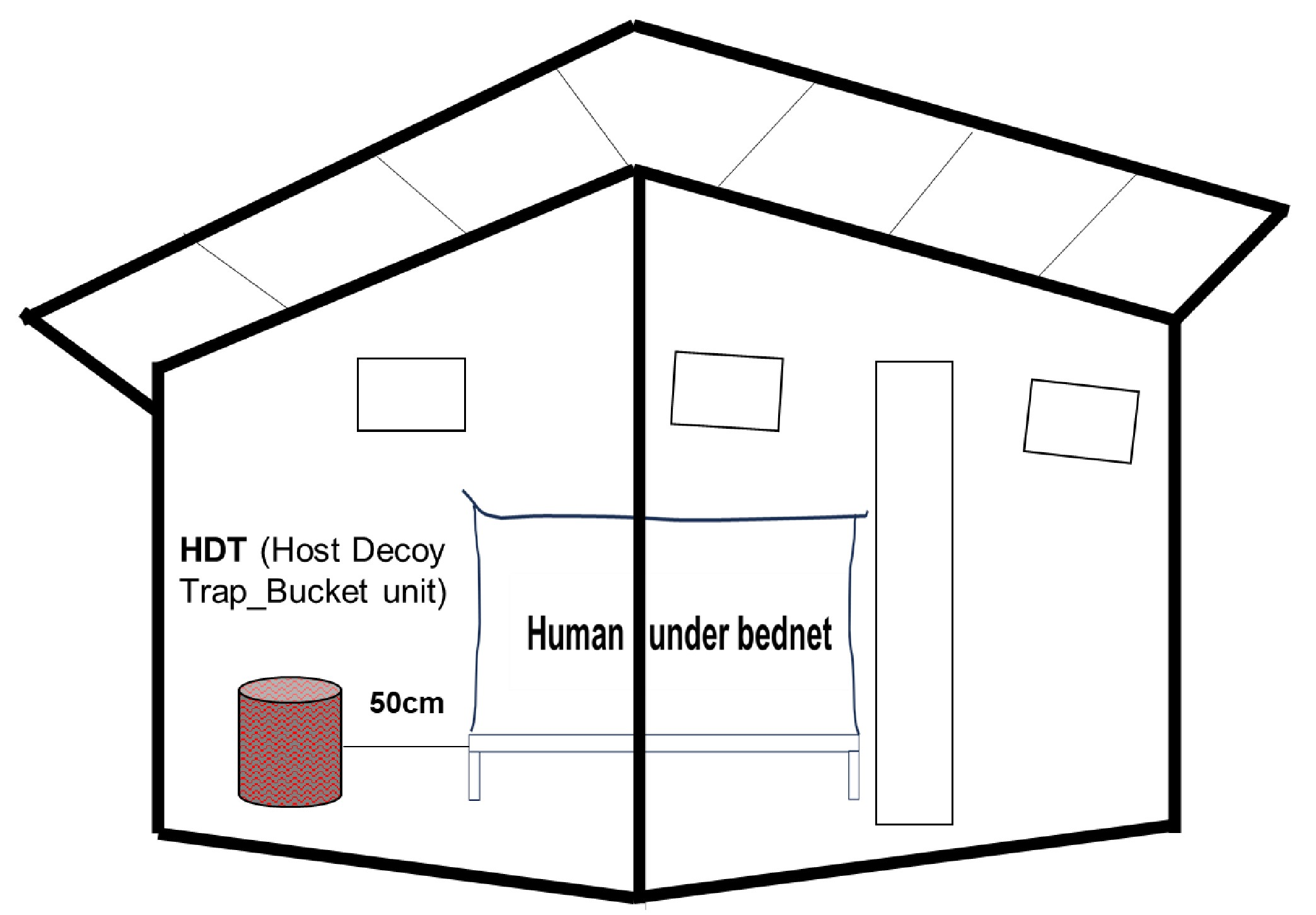

Supplement: Supplementary file 1 — Additional file 1: Experimental set-up schematic of HDT with person under a bednet inside an experimental hut. [file 13071_2025_6759_MOESM1_ESM.tiff]
